# Detecting and Correcting for Human Obstacles in BLE Trilateration Using Artificial Intelligence

**DOI:** 10.3390/s20051350

**Published:** 2020-02-29

**Authors:** Sharareh Naghdi, Kyle O’Keefe

**Affiliations:** Position, Location and Navigation (PLAN) Group, Department of Geomatics Engineering, Schulich School of Engineering, University of Calgary, 2500 University Drive, N.W., Calgary, AB T2N 1N4, Canada; sharareh.naghdi@ucalgary.ca

**Keywords:** trilateration, BLE, artificial intelligence, localization, obstacle

## Abstract

One of the popular candidates in wireless technology for indoor positioning is Bluetooth Low Energy (BLE). However, this technology faces challenges related to Received Signal Strength Indicator (RSSI) fluctuations due to the behavior of the different advertising channels and the effect of human body shadowing among other effects. In order to mitigate these effects, the paper proposes and implements a dynamic Artificial Intelligence (AI) model that uses the three different BLE advertising channels to detect human body shadowing and compensate the RSSI values accordingly. An experiment in an indoor office environment is conducted. 70% of the observations are randomly selected and used for training and the remaining 30% are used to evaluate the algorithm. The results show that the AI model can properly detect and significantly compensate RSSI values for a dynamic blockage caused by a human body. This can significantly improve the RSSI-based ranges and the corresponding positioning accuracies.

## 1. Introduction

Application of Bluetooth Low Energy (BLE) technology for indoor positioning systems has recently gained considerable attention due to the low power and ease of deployment of BLE beacons. Radio-based indoor positioning, including BLE positioning, generally can be divided into three broad categories: proximity, Time of Flight (TOF) measurements, and Received Signal Strength Indicator (RSSI) based methods. In some cases these have also been augmented with Angle of Arrival (AOA) [1]. RSSI-based ranging and fingerprinting are two common methods that rely on the RSSI measurements for positioning. Positioning using BLE fingerprinting has the potential to achieve high accuracy provided sufficiently dense training data are available, however, this process is time-consuming and does not adapt to environmental changes. BLE RSSI-based ranging uses a path loss model to estimate ranges from RSSI values and then trilateration to compute a position.

RSSI-based ranging, like fingerprinting, also requires accurate calibration using RSSI measurements to estimate the path loss model and, again, is sensitive to changes in the propagation environment. The RSSI and the path loss between the transmitter and the receiver are often modelled assuming a 1/n model:

(1) $$RSSI=RSSI\left(d_{0}\right)-10n{log}_{10}(\frac{d}{d_{0}})$$

where *RSSI*(*d*_0_) represents the RSSI value at the reference distance *d*_0_, *n* is the path loss exponent value, and *d* is the distance between the transmitter and receiver. In free space, *n* is 2 while it often is greater due to other sources of attenuation, and can be less than 2 in wave-guides.

BLE uses three channels for advertising and 37 channels for the transmission of data. Because each channel has a slightly different carrier frequency, each BLE advertising channel will have different power level due to differing channel gain and multipath fading.

**Figure 1:**
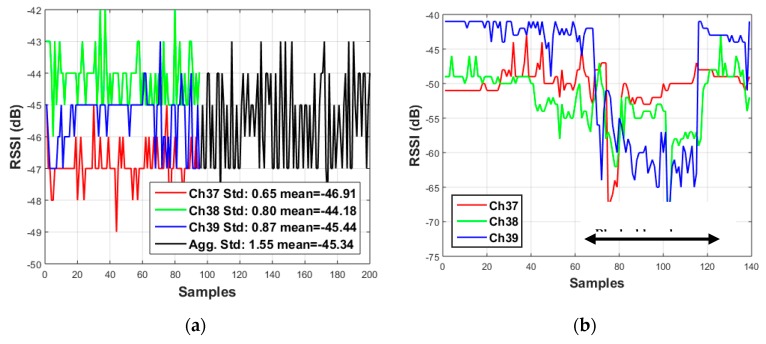
(**a**) Received Signal Strength Indicator (RSSI) samples received from the three advertising channels compared to combination (aggregate). (**b**) Effect of blockage on the RSSI samples received from the three advertising channels.

Many proposed BLE RSSI-based ranging and fingerprinting systems often use RSSI from all three advertising channels together to obtain an aggregate signal [2,3,4]. However, the signals have been shown to exhibit different overall path-loss models and are affected by multipath fading and attenuation differently. As a result, considering all channels together (aggregate) can give the appearance of large fluctuations in power level (from channel to channel) even when the individual channels’ power levels are stable. Furthermore, one or more channels may experience multipath fading while the others do not. An example of this is shown in Figure 1a, where the first 100 samples of each channel are used to compute different means with small standard deviations, while next the 100 samples are plotted in aggregate mode resulting in a single mean value with a large standard deviation. When the channels are considered separately, small fluctuations are visible in each, however these are lost when considering them in aggregate.

Reference [5] proposed fingerprinting using per-channel training data while several recent works have used individual channel data for RSSI-based trilateration. In [6] the authors make use of the diversity of BLE channels in an algorithm combining a polynomial regression model, fingerprinting, two levels of outlier detection, and extended Kalman filtering, however, the investigated scenario was an empty corridor and the effect of obstacles was not investigated. Another recent study is presented in [7], where channel information, a Kalman filter and a trilateration method are applied to improve the precision of BLE RSSI-based trilateration. In this case, one or more mobile users were transmitting and several stationary receivers were deployed where each receiver was equipped with separate hardware to monitor each of the three advertising channels in parallel and then only the single channel with the least variation on each link was then used for trilateration. A robust positioning system based on separate channels was investigated in [8]. Unlike [6,7,8] it begins by using training data to fit a path-loss model to each channel. Then in the operational stage, RSSI observations are taken and median values are used to estimate ranges for each channel. For each link, a weighted average range is then assembled from the ranges obtained from the available channels. This average range is then used for trilateration. Our previous work [9] has also shown that the using all three advertising channels, each with their own empirically determined path loss exponent, can provide more accurate results than using all available RSSI observations in aggregate with a single path loss mode.

In wireless propagation, fading may either be due to interference from multipath propagation or shadowing from obstacles. Due to the difficulty of modeling the signal attenuation caused by obstacles, this effect is often neglected in the literature. However, to properly model radio propagation for RSSI-based ranging, all of the obstacles between the transmitter and receiver should be considered. A common signal attenuation source is the human body which can shadow or fully obscure the signal path. Figure 1b shows an example where multipath fading can be seen on channel 38 between samples 40 and 60 while all three BLE advertising channels are attenuated by a human body blockage beginning near sample 60.

There are a variety of studies that investigate the effect of human bodies on fingerprinting-based and range-based positioning [10,11,12]. Early work approximated the attenuation due to body shadowing with a Rician distribution [10]. A path loss shadowing model for RSSI-based ranging based on a Markov process was introduced in [11], where shadowing effects on each propagation path were generated individually using a transition probability. In RSSI-based fingerprinting, training data are typically collected for multiple orientations to compensate for attenuation due to the person holding the mapping device. [12] showed that the signal distribution with distance takes an elliptical shape due to the presence of a human body. Then, based on the properties of the ellipse, a signal attenuation model was proposed to generate an orientation-independent fingerprinting database. Although the above mentioned works have given a comprehensive analysis of the effects of body shadowing and body orientation on RSSI-based positioning, none of these works introduced a dependency of shadowing on the distance of a body from a transceiver, nor did they consider the influence of the number of bodies. However, there are some studies that consider the number of people or their distances from the transmitters in accounting for the shadowing effects on the propagation model [13,14,15]. A twin cylinder model for moving human body shadowing at 60 GHz that considers the geometrical position of a human body was presented in [13]. Indoor radio channel characterization research to detect and characterize the motion of network nodes and moving objects in a network environment using the changes in RSSIs is presented in [14]. RSSI mean and variance values can be useful indicators in signal-based human detection techniques. There are many algorithms to monitor mean and variance of RSSI for detecting the presence of human bodies and other obstacles [15].

In our preliminary work [9], we proposed to exploit the fact that obstacles cause attenuation on all three channels simultaneously while multipath affects the channels individually. We suggested a BLE thresholding-based technique where the reduction in current RSSI sample is compared with the mean of a set of previous samples. If the new RSSI is attenuated by one standard deviation below the mean RSSI in that channel, and all the three advertising channels have a similar attenuation pattern, a detection event is declared.

All of these models ([9,10,11,12,13,14,15]) are computationally efficient and easy to implement, though less able to tackle the sudden changes in the real environment. Unlike analytical or empirical models, Artificial Intelligence (AI) networks can learn from observed data [16,17,18] in real environments and identify patterns that might not be captured by rule-based thresholding techniques. AI is particularly useful when the correlation between the input and output values of a system is ambiguous or subject to noise [19].

AI techniques such as neural networks can be utilized as effective methods to achieve high computational efficiency and robustness against noise and interference in indoor positioning techniques. The majority of the previous studies use AI models as tools to determine sensor locations based on general parameter information such as RSSI [5] and transmitter identification in advance [20]. Other AI studies determine the BLE RSSI values for indoor environments [21] using more specific information such as direct distance between transmitters and receivers to optimize the RSSI values. In [22] an RSSI real-time correction method based on the particle a swarm optimization—back propagation neural network is proposed, however, the method needs a gateway to collect RSSI in real time on top of the receiver measurements.

In this paper, we propose AI models to detect and compensate the obstacle shadowing effect in a dynamic indoor environment using the three BLE advertising channels. Figure 1b shows that how the three BLE advertising channels follow the same pattern in case of human body blockage. When a human body blocks the signal all three channels drop and when the human body leaves the area all three channels return to the former values. Using the advantage of this uniform reaction of the signals to the blockage lets the AI algorithm to distinguish between multipath fading and human body showing. Demonstrating the effectiveness of this strategy is the main contribution of this study. To our knowledge, there is no prior research using AI algorithms to model and enhance RSSI values due to human body shadowing by taking advantage of the three BLE advertising channels. Unlike thresholding-based techniques, our proposed algorithm applies a sliding window to compare all channels inside the AI model. The sliding window technique gives the opportunity of learning sequential patterns of RSSI to predict a possibility of human body shadowing. Moreover, the present research is evaluated in terms of a trilateration positioning solution that provides the opportunity to reduce the error by predicting propagation behavior of radio signals properly accounting for human body shadowing using the three separate BLE advertising channels.

The remainder of this paper is organized as follows: Section 2 focuses on the methodology of the algorithm and theoretical basis of the system. Section 3 evaluates the algorithm with various field experiments. Finally, the effectiveness of the method is validated and discussed in Section 4.

## 2. Methodology

The proposed method uses an artificial neural network to identify patterns in the RSSI observed on the three BLE advertising channels. The premise is that while the three channels will have different values and these values will vary differently due to frequency dependent fading, when an obstacle, such as a human body, is present, it should affect all of the channels simultaneously. Rather than setting a detection threshold, we propose to train an artificial neural network to learn to identify the patterns corresponding to shadowing due to human bodies.

### 2.1. BLE and Channel Hopping Overview

BLE is a wireless communication technology that works in the 2.4 GHz frequency Industrial Scientific and Medical (ISM) band with a total of 40 channels, each one 2 MHz wide. For discovery services, it uses three advertising channels: 37 (2402 MHz), 38 (2426 MHz) and 39 (2480 MHz). In this work, the receiver was configured to separately record the RSSI values observed on all three advertising channels. While each advertising channel has different RSSI values, noises and fading, the RSSI values on all three channels attenuate when obstacles are present.

### 2.2. Human Body Detection

**Figure 2:**
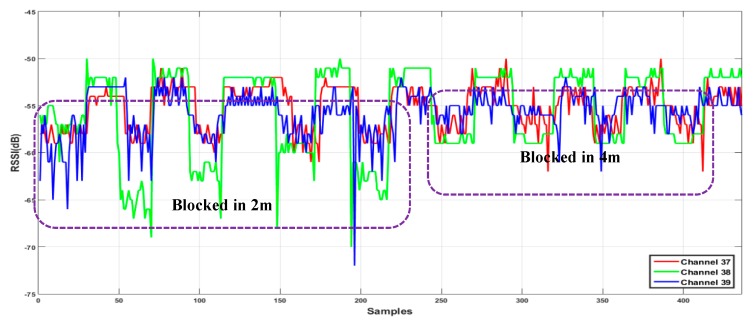
Repeatable response of three Bluetooth Low Energy (BLE) advertising channels due to the human body.

The human body is a source of an additional propagation loss. In this paper, the similar response of the three BLE advertising channels to obstruction by a human body is used to detect and compensate the RSSI values for this effect. In particular, Figure 1b shows an obvious example of this kind of detection based on three channels. To ensure the repeatability of the attenuation due to human body obstruction repeated RSSI measurements were made over a 6-meter distance between a transmitter and receiver and a test subject was made to periodically obstruct the signal. During 450 RSSI measurement epochs, the test subject blocked the line-of-sight 2 m from the receiver five times and then moved and block the line-of-sight four additional times, this time 4 m from the receiver. The RSSI time series of this test is shown in Figure 2. All nine obstruction events are evident, however the magnitude of the effect is smaller when the obstacle is farther from the receiver. Based on these results, we have assumed that the human obstacle will be detectable using this method when it is close enough to the receiver to affect the RSSI. These tests were done in simple environments and obviously real indoor environments will be more complicated and require additional testing.

### 2.3. Artificial Neural Networks (ANNs)

AI techniques can be used to process complicated and noisy input because with sufficient training data AI can learn patterns that are not obvious to conventional decision-making techniques. In this paper, a fixed number N of past BLE RSSI values are used as inputs to an Artificial Neural Network (ANN). The goal of the N samples in the sliding window technique is to detect the moments that human body blocks the signal or gets out of the way. To see this effect, the set of samples needs to be just long enough to detect the transition. If the sequence is too short then no pattern will be detectable while too long a sequence will require the ANN to also learn to deal with multiple transitions, which we wanted to avoid. We chose 10 samples as the window length based on this reasoning.

These inputs may or may not be affected by human body shadowing. The ANN outputs include three corrected RSSI values (one for each channel) and the blocking information state (that an obstacle is present or not). The output RSSI values represent the RSSI values corrected for the ideal situation with no shadowing. During the training phase, RSSI values with and without human body obstruction were provided for input, however, the corresponding outputs for training were sampled from only the set of RSSI values with no obstructions observed from the same receiver position. This approach was selected rather than evaluating the path loss model for that range so that the corrected RSSI values would have a similar variance to real line-of-sight RSSI values.

Several forms of ANN are often applied to signal processing problems. These include the Multi-layer Perceptron (MLP) model, the Radial Basis Function (RBF), and the Support Vector Machine (SVM) [19]. SVM outperforms MLP and RBF in classification problems, although, for regression-based problems SVM generally achieves lower accuracy [23]. A comparison between MLP, RBF and SVM models to predict a time series model has been presented in [24] where it was concluded that the MLP and RBF models performed better than SVM in time series problems and MLP and RBF were selected for this work for this reason.

#### 2.3.1. Multi-Layer Perceptron (MLP)

This paper applies a supervised MLP learning method with error back propagation. In the back propagation algorithm the input vector is propagated with fixed weights and biases through a forward pass and the output is produced. Then synaptic weights and biases are adjusted by using the error signal that propagates backward to minimize the cost function of the neurons in the output layer:

(2) $$C=\frac{1}{2m}\overset{m}{\underset{i=1}{\sum }}{\left||\left(\tilde{y}\left(x_{i}\right)-{\tilde{y}}^{l}\left(x_{i}\right)\right)|\right|}^{2}$$

where $\tilde{y}\left(x_{i}\right)$ and ${\tilde{y}}^{l}\left(x_{i}\right)$ represent the desired output and the actual output, respectively. $x_{i}$ represents the ith training example. *l* and *m* denote the number of layers and the number of training examples, respectively.

#### 2.3.2. Radial Basis Function (RBF)

RBF is an ANN technique that identifies the activation of a hidden unit by the distance between the input vector and a prototype vector during the training. Each neuron in the hidden layer consists of a radial basis function and the output layer is a weighted sum of the outputs from the hidden layer. The hidden and output layers apply a nonlinear and a linear transformation, respectively. The training procedures in RBF networks can be significantly faster than the training procedures in MLP networks. There are two stages in the training procedure of a RBF network. The first stage involves the determination of the mean value and distance from the center of the activation function using the input data by unsupervised training methods. In the second stage, the output layer weight vector is determined. In RBF, the hidden layer uses a set of Gaussian functions given by:

(3) $$\varphi \left(x,\mu \right)=exp\left(-\frac{{\left(x-\mu \right)}^{2}}{2d^{2}}\right)$$

known as radial basis functions, where μ is the center of Gaussian function (i.e., the mean value of x) and *d* is the distance from the center of the Gaussian function. The output of each hidden unit is based on the distance of the input from the center of the Gaussian radial function φ(x,μ). Then, data points closer to the center of the radial basis function have more effect on the results. This effect can be adjusted by controlling the distance (*d*). Parameters (*d*) and (μ) are defined and adjusted separately at each RBF unit during the training procedure. Layer 3 or the output layer is a weighted linear combination of the outputs from the hidden layer:

(4) $$output=\sum_{i}\left(\varphi_{i}W_{i}\right)$$

### 2.4. Distance Model and Position Estimation

A standard log-distance path loss model has been selected to convert RSSI to distance as shown in Equation (1). Usually parameter $d_{0}$ is fixed to 1 meter, and $RSSI\left(d_{0}\right)$ is the average measured RSSI when the receiver is 1 meter away from the transmitter as is required in all logarithmic models. The path loss exponent (*n*) is related to the wireless environment and it and $RSSI\left(d_{0}\right)$ can be determined either by fitting a line to training measurements, or by choosing standard values. Theoretically, the *n* should be fixed, however, in reality, the BLE transmit power has time-varying characteristics, and the path loss exponent is dependent on the environment. As a result it is difficult to identify the relationship between RSSI and the distance accurately when applying the log-distance model. Researchers have used different techniques to fit the RSSI distance model more accurately such as additional gateway corrections [19], or adding random noise [6,25].

In this paper, the log-distance parameters for each channel were determined empirically from the training data in an environment without any obstructions, but performance will also be evaluated using standard (non-empirical) values.

Where this paper differs from prior work is that ANN and a training data set are used to correct the RSSI values that have been affected by obstructions leading to more accurate range estimation compared to prior studies.

Finally, the corresponding direct distance from each transmitter to the receiver is calculated for each channel by using the corresponding log-distance model and the RSSI values that may have been corrected by the AI. All available ranges are then used in the trilateration using non-linear parametric least-squares. If *m* transmitters with known coordinates $(x_{Tx_{1}},y_{Tx_{1}}),\;\left(x_{Tx_{2}},y_{Tx_{2}}\right),\ldots ,\;\left(x_{Tx_{m}},y_{Tx_{m}}\right)$ are deployed, and the receiver has unknown location $\left(x_{Rx},y_{Rx}\right)$, the m distances are related to the unknown positions as

(5) $$d_{m}=\sqrt{{\left(x_{Tx_{m}}-x_{Rx}\right)}^{2}+{\left(y_{Tx_{m}}-y_{Rx}\right)}^{2}}$$

This observation model must be linearized to obtain the design matrix (*H*) which contains information regarding the geometry of the measurements.

(6) $$H_{m}={\left[\begin{array}{cc} \frac{-\left(x_{Tx_{m}}-\;x_{\mathrm{Rx}}\right)}{d_{m}} & \frac{-\left(y_{Tx_{m}}-y_{Rx}\right)}{d_{m}} \end{array}\;\right]|}_{x\;=\;\hat{x}}$$

To make this distinction more explicit, the state vector $X={\left[x_{Rx}\;y_{Rx}\right]}^{T}$, is estimated using a set of observations *z* to minimize the sum of squares of residuals, z−h(X), where h(X) is the non-linear form of the observation model.

### 2.5. Implementation

**Figure 3:**
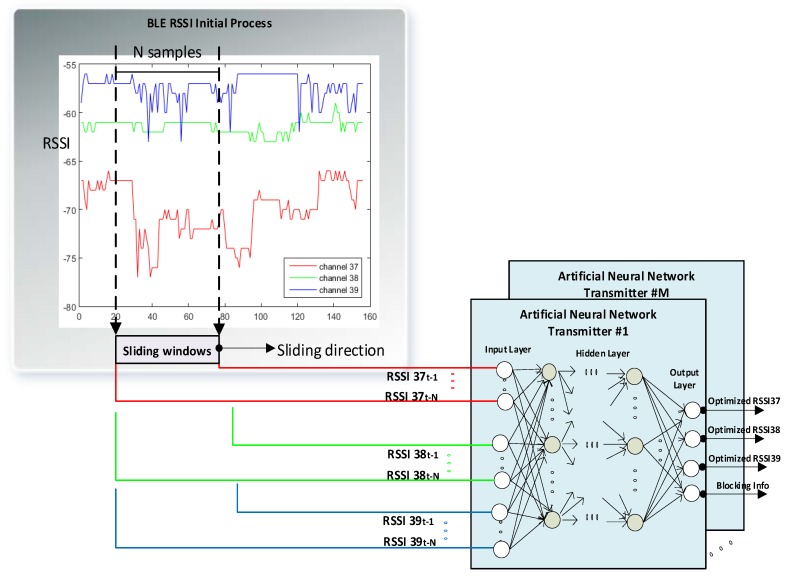
General block diagram of detecting and compensating the obstacle shadowing effect in a dynamic indoor environment using the three BLE advertising channels.

Both the MLP and RBF ANNs are implemented similarly. For each transmitter, *N* previous RSSI samples from each channel fed as input into the ANN structure (Figure 3). The output of the ANN system consists of the optimized RSSI values and the blockage state. Output RSSI values for training are obtained from an additional calibration data set with no obstructions that was also used to determine empirical path loss exponents for each transmitter.

**Figure 4:**
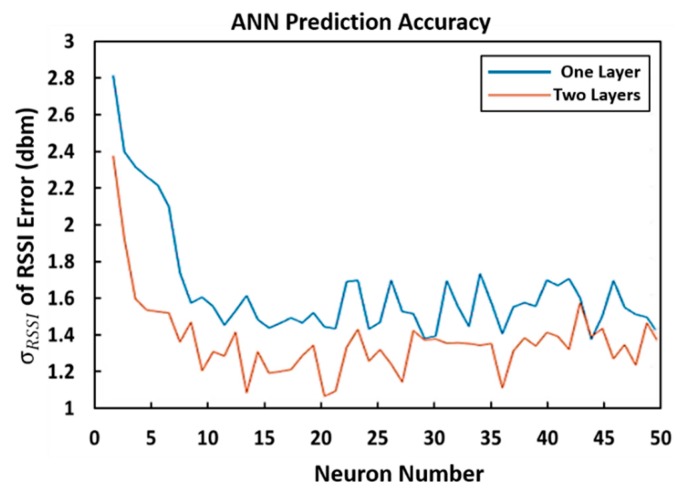
Standard deviation of output errors of Multi-layer Perceptron (MLP) in various layers and neurons.

To design the MPL, different numbers of hidden layers (1 and 2) with different numbers of neurons (1 to 50) were investigated. Figure 4 shows the relationship between the total number of neurons and standard deviation ($\sigma_{RSSI}$) of the compensated RSSI errors. Two hidden layers generally provided more accurate prediction solutions than one. There is also no improvement in prediction accuracy beyond 20 neurons per layer. As a result, an architecture with two hidden layers and 20 neurons was adopted for MPL and a hidden layer and 20 neurons was adopted for RBF.

## 3. Experimental Setup

In order to train, validate, and test both ANNs, two data collections were conducted in an empty medium size room at the University of Calgary with dimensions of approximately 6 m × 11 m. We deployed four BLE transmitters, one on each wall with known locations. Reference points for the receiver with one meter spacing were marked on the floor. The transmitters were DWM1001-DEV (Decawave Ltd., Dublin, Ireland) modules that include a DW1000 UWB chip (Decawave Ltd., Dublin, Ireland), a nRF-52832 (Nordic Semiconductor, Trondheim, Norway) BLE radio [26], and an accelerometer. The BLE advertising information was sent with an interval of 20 ms. In the first test, shown in Figure 6c, the test subject held a nRF52840 DK (Nordic Semiconductor, Trondheim, Norway) [27] BLE module in front of her body to measure the RSSI values on all advertising channels with a scan interval of 50 ms.

Here, a passive and connectionless scan was executed where the transmitters are not aware of how many advertising packets were actually received by the receiver. During each 50 ms scan interval the receiver is measuring one channel and will log RSSI values that are available during that interval. The receiver switches channels every scan interval [28,29].

1500 samples of RSSI were collected from each transmitter on each of the three channels. This dataset included 750 line-of-sight epochs and 750 where one of the four lines-of-sight is obstructed by a second test subject who obstructed the line-of-sight between transmitter #4 and the receiver at a random distance between 1 and 3 m from the receiver. 10 reference points with approximately 1 meter spacing in a line between transmitters #2 and #4 were occupied. The receiver was moved to each reference point to take 150 RSSI measurements (75 with obstruction and 75 line-of-sight). It should be noted that in “unobstructed” samples, the line-of-sight from transmitter #2 to the receiver is always obstructed by the test subject holding the receiver.

**Figure 5:**
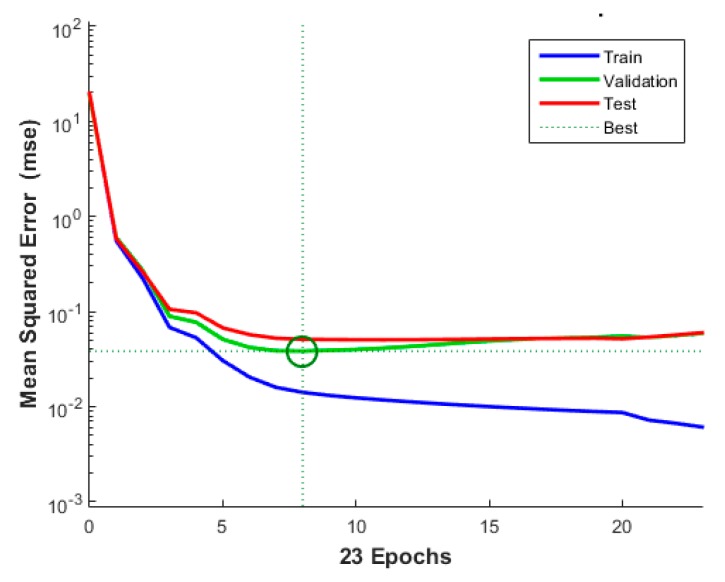
Train, Validate and Test performance for transmitter number 4, best validation performance is 0.038404 at epoch number 8.

Then from 750 measurements in obstructed case, 525 have been employed to train the network, 75 for validation purposes and remaining 150 non-training observations used to test its performance. The same breakdown of the 750 line-of-sight measurements was to use for training, validation and testing. Figure 5 shows the training, validation and testing performance for observations from transmitter #4 in terms of mean squared error (in RSSI) as a function of the number of iterations. The training stop criteria was chosen from the point where the validation data reached a minimum error. The model is able to converge within eight iterations and model weights are chosen based on this epoch.

**Figure 6:**
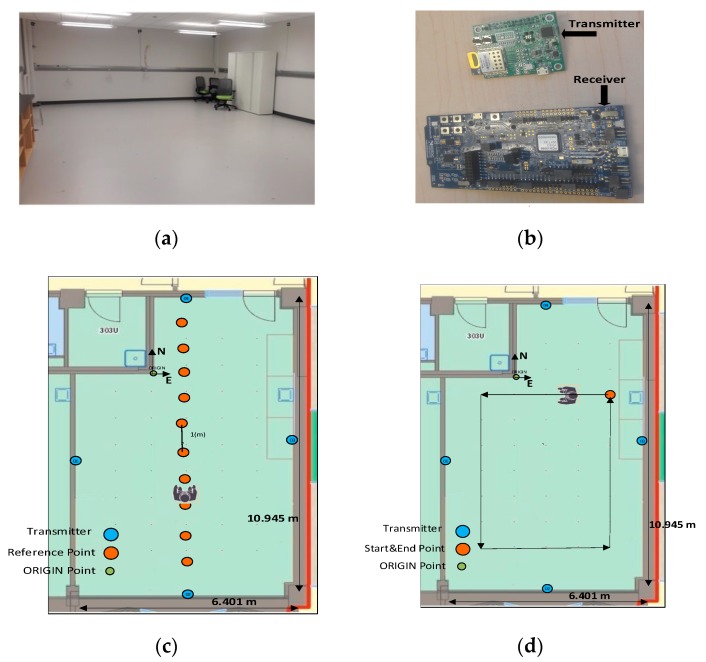
(**a**) Test environment (**b**) Sensors (**c**) General block diagram of detecting and compensating the obstacle shadowing effect in a dynamic indoor environment using the three BLE advertising channels test case #1 and (**d**) test case #2.

In case 2, RSSI values were measured while a closed-loop trajectory was walked continuously with the operator holding the receiver on her head as shown in Figure 6d. The loop was repeated twice: once with no obstructions, and once with a second test subject walking the same trajectory one meter ahead of the test subject. In both cases, 70% of the data collected was selected randomly for training and the remaining data were used for testing.

## 4. Results and Discussion

The ability of both MLP and RBF to correct the RSSI values for human body shading was evaluated in the range domain using the log-distance model with empirical path loss exponent to convert RSSI distance estimation. The AI-based results were compared to the method used in [25], referred as the classic log-distance method, where the same empirical path loss exponent was used, but a constant shadowing loss is applied to all measurements to account for possible obstruction. On the other hand, to see the effect of the empirical model in the proposed method, all three methods (classic, MLP and RBF) tested with a fixed (non-empirical) path loss exponent set to *n* = 2.5 for all channels. This number was selected based on other studies for indoor areas that used path loss exponents between 2.4 to 2.6 [30,31].

**Figure 7:**
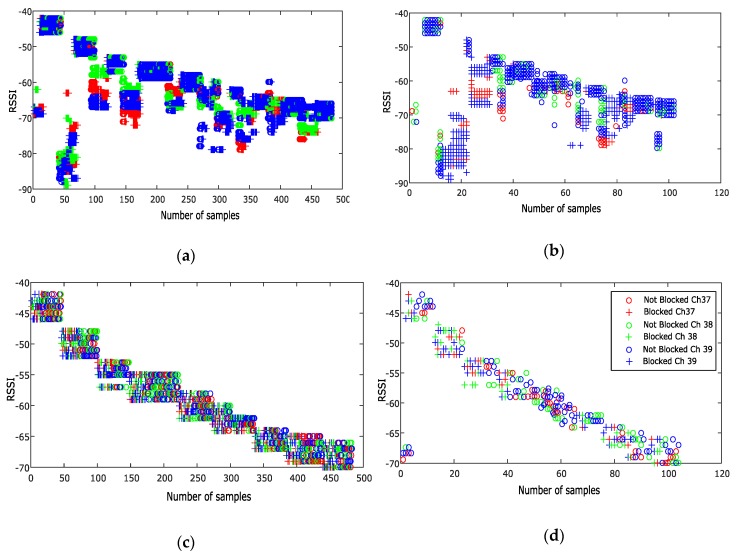
Blocked and not blocked RSSI values in MLP algorithm from transmitter #4 in channel 37, 38 and 39, for input training (**a**), output training (**c**), input test (**b**) and output test (**d**) (All RSSI value are presented in dB).

The results of the MLP algorithm on the RSSI values for transmitter #4 in the training and test data sets are presented in Figure 7. RSSI values of all three advertising channels were compensated during human body shadowing events that occur around samples 20 and 80 in the test data. In these cases the plus symbols, representing the observed RSSIs, were consistently low and the ANN was able to distinguish and compensate the shadowing effect in the output (Figure 7d). However the MLP is not perfect and generates both missed detections and false alarms. For example, it occasionally misclassified fading on one or two channels as blocking and corrected the RSSI values (while this was a classification error, was beneficial as the result was a corrected RSSI). In addition, in the first three samples of the test data, that were experiencing fading on all channels, it was unable to recognize this as there was no transition in the time series from full power to faded, resulting in an uncorrected RSSI that is then interpreted as a much larger distance. Table 1 represents the numbers of corrected and uncorrected RSSI samples and how they were classified by both the MLP and RBF methods. As discussed in Section 3, 300 test epochs in test cast #1 (150 obstructed and 150 unobstructed). Each contains RSSI measurement from the four transmitters for a total of 1200 observations (600 obstructed and 600 unobstructed samples).

MLP was able to correctly classify 89% of the epochs in the test data where human body shadowing was present while 11% were missed detections. When shadowing was not present, only 6% of these epochs were false alarms while the remaining 94% were correctly classified as line-of-sight situations. RBF performed slightly worse with 13% missed detections and 8% false alarms.

The results were compared in terms of the range and position errors in both test cases:

### 4.1. Test Case #1

**Figure 8:**
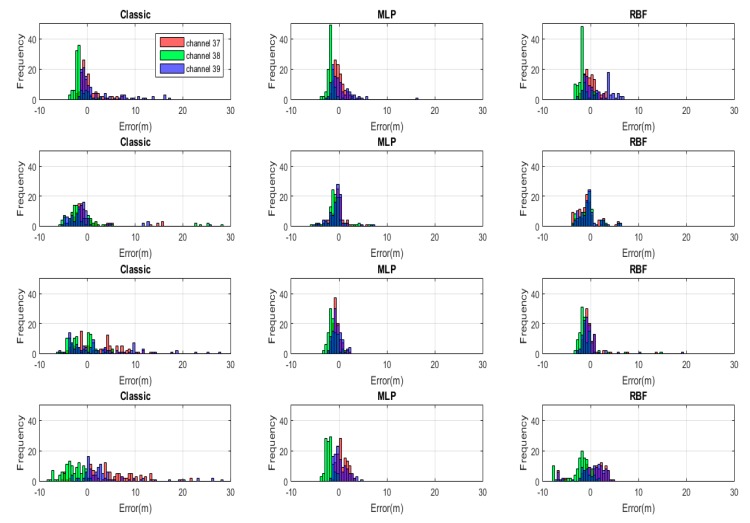
The histogram of distance estimation errors for all the transmitters (each transmitter presented in one row) by using three different methods (classic, MLP and Radial Basis Function (RBF)) for each BLE advertising channel with empirical model.

**Figure 9:**
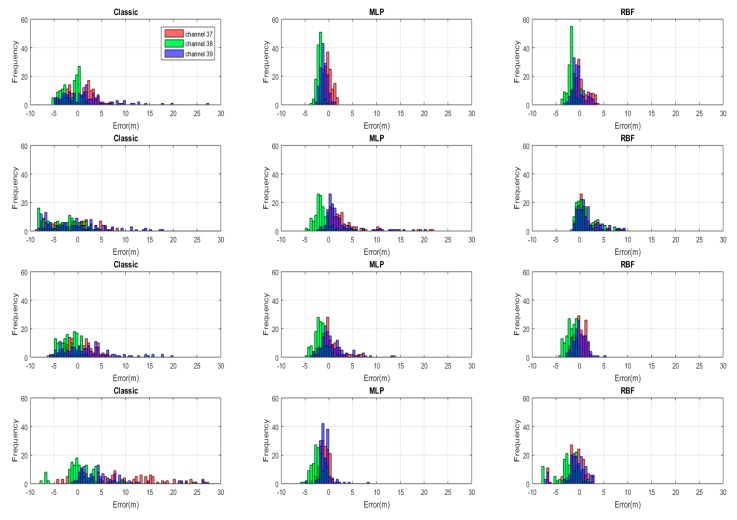
The histogram of distance estimation errors for all the transmitters (each transmitter presented in one row) by using three different methods (classic, MLP and RBF) for each BLE advertising channel with non-empirical model.

Figure 8 exhibits the range error distribution by histograms for different BLE channels, using three different methods (classic, MLP, and RBF) and empirical path loss exponents, and four different transmitters, while, Figure 9 represents the same results with non-empirical models. When the classic log-distance algorithm with empirical path loss was used, the majority of absolute range errors were widely spread in less than 10 m in all directions. However, in both ANN methods, MLP and RBF with empirical path loss model, the range error distribution tended to concentrate around zero with spread of less than 2.5 m. Applying the empirical path loss exponent improved the classic results, but barely changed MLP and RBF ranges. All the histogram standard deviations in both figures (Figure 8 and Figure 9) are summarized in Table 2.

**Figure 10:**
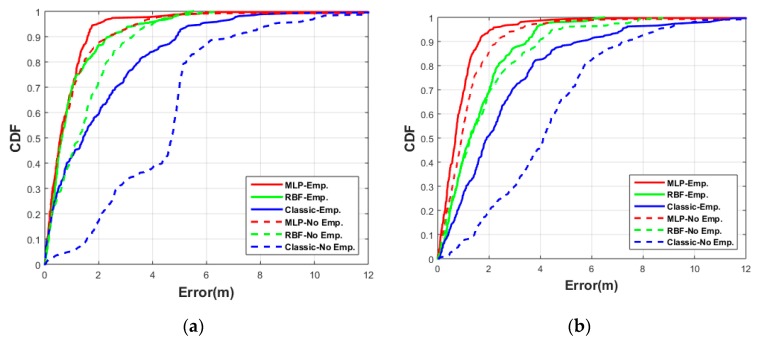
Test #1 cumulative distribution functions (CDFs) of absolute East-West position error (**a**), and North-South position error; (**b**) using the three different methods (MLP, RBF, and Classic) with empirical path loss exponent (solid lines) and with fixed path loss exponent = 2.5 (dashed lines).

In addition, Figure 10 presents the cumulative distribution functions (CDFs) of absolute values of the horizontal position error components (East-West and North-South) for the three different methods with empirical and non-empirical path loss exponents. The East-West position error with an empirical path loss was better than 1.6 m for 90% of the samples when using the MLP method, which is a 67% improvement compared to the classic log-distance with a shadowing factor (4.8 m). The RBF method also showed acceptable position error (less than 2.4 m) or an improvement of 50% compared to the classic log-distance method. The position error in the North-South direction showed similar improvement with the 90% localization error for MLP at 1.7 m and for RBF at 3.5 m, which are 68% and 34% improvements over log-distance (5.3 m), respectively.

The position error using the non-empirical path loss in the East-West direction was better than 2.2 m 90% of the time using MLP method, which is a 67% improvement compared to the non-empirical classic log-distance method (6.7 m). The RBF method also showed acceptable position error (less than 3.2 m) or an improvement of 52% compared to the non-empirical classic log-distance method. The AI-supported results were very close to those using the empirical path-loss model. The position error in the North direction with non-empirical also showed similar improvement with the 90% localization error for MLP at 2.3 m and for RBF at 4 m, which were 68% and 45% improvements over non-empirical log-distance (7.3 m), respectively. It should be noted that the classic method using the non-empirical path-loss exponent suffered in the East-West direction compared to the classic results using the empirical path-loss because the classic method applied a shadowing loss to all observations and in the test scenario the observations to transmitters 1 and 3 were never obstructed.

### 4.2. Test Case #2

The corrected RSSIs in the second test set were measured by walking inside a closed loop with approximate size of 4 m × 5 m (Figure 6d).

**Figure 11:**
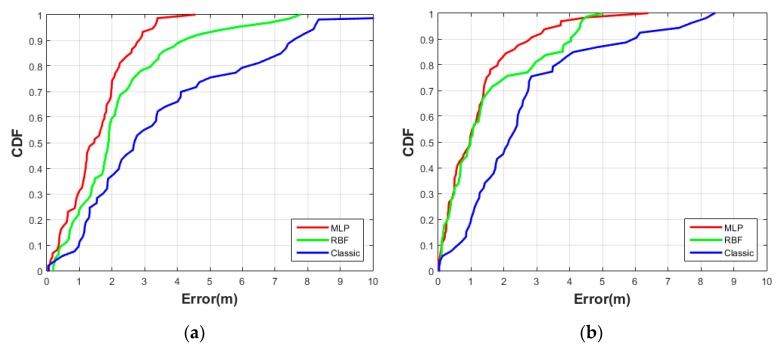
Test #2 CDFs of absolute East-West position error (**a**), and North-South position error; (**b**) using the three different methods (MLP, RBF, and Classic).

Figure 11a depicts the CDF of position error in the East direction. Both ANN models (MLP and RBF) show positioning error about 2.7 m to 4 m compared with a classic log-distance method which shows 7.4 m in 90% the data. MLP method is decreased by 64% and RBF method 46% over the log-distance method. In Figure 11b the positioning errors in the North direction represent 2.8 m to 4 m positioning error in 90% for both ANN models (MLP and RBF), while the log-distance model shows more error (6 m).

## 5. Conclusions

In this paper, we have proposed and implemented a sliding window ANN method for detecting human body attenuation in BLE RSSI values using the three advertising channels. The method uses a sliding window of RSSI input from all three advertising channels to distinguish attenuation due to sudden obstacle blockage versus fading due to multipath or gradual signal level change due to changing range and then corrects the RSSI level to match those for the unblocked case. Both ANN methods are able to correctly identify sudden blockages more than 87% of the time. The corrected RSSI values are then converted to ranges using a simple log-distance model with empirical path loss exponents also obtained from the training data and compared to those obtained using a textbook value. The ranges are used to compute position through trilateration. Our results show significant improvement in range and position accuracy compared to a previously proposed method where all RSSI measurements were adjusted for shadowing, whether it occurs or not.

It is interesting to compare our results to those reviewed in the literature. Reference [7] shows 1.82 m accuracy 90% of the time in a 6 by 9 m room and 4.6 m in a 16 by 17 m room, results which are close to ours (2.3 m in scenario #1 and 3.8 m in scenario #2, 90% of the time). Reference [6] achieved accuracies of less than 2.56 m 90% of the time, (an average of two trajectories) with a dense deployment of BLE beacons (one beacon per 9 m) but did not investigate obstacles. Finally reference [8] shows a positioning accuracy of less than 2.4 m 90% of the time using a differential correction method that requires a nearby reference receiver with known coordinates that experiences the same obstruction.

Since real environments are more complicated than those tested in this study and other papers, with real traffic and multiple obstructions, training the ANN to identify multiple human obstructions is one of the most important areas for future investigation. We hope to investigate the use of vision technology as an addition source of information about the presence of obstacles attenuating BLE RSSI while expanding our tests to more diverse indoor environments with other types of static and dynamic obstacles.

## Figures and Tables

**Table 1:** Artificial Neural Network (ANN) performance in terms of correct detection, missed detection and false alarm.

Status	Samples in MLP ^1^		Samples in RBF ^2^	
Correct detection (obstruction)	534	89%	522	87%
Missed detection	66	11%	78	13%
False alarm	36	6%	48	8%
No detection (no obstruction)	564	94%	552	92%

^1^ Multi-layer Perceptron (MLP). ^2^ Radial Basis Function (RBF).

**Table 2:** Standard deviation of range error (all in meters).

TX ^1^s	Algorithm	Channel 37	Channel 38	Channel 39
TX1	Classic	2	1.2	4.6
	MLP	1	0.7	2.3
	RBF	1.3	1	2.5
	Classic-Non Emp ^2^.	3	2	5
	MLP-Non Emp.	1	0.8	2.3
	RBF-Non Emp.	1.4	1	2.3
TX2	Classic	4.2	6.5	4.4
	MLP	1.7	2	1.7
	RBF	2	2	2
	Classic-Non Emp.	4.3	7	6.8
	MLP-Non Emp.	2.3	2	2.5
	RBF-Non Emp.	2.1	2	2.2
TX3	Classic	4.6	2.5	8.8
	MLP	0.8	0.8	0.9
	RBF	1.8	2	2.4
	Classic-Non Emp.	5.5	4	9
	MLP-Non Emp.	2.3	1.2	2.5
	RBF-Non Emp.	1.9	2	2.3
TX4	Classic	4.6	2.5	8.8
	MLP	0.8	0.9	0.9
	RBF	1.8	2	2.1
	Classic-Non Emp.	8	3.2	11
	MLP-Non Emp.	0.9	1.1	1.3
	RBF-Non Emp.	2.1	2.1	2.1

^1^ Transmitter (TX). ^2^ Empirical (Emp).
